# Computer-aided design enables repurposing of proprotein convertase subtilisin/kexin type 9 degrader from cholesterol-lowering to colon cancer therapy

**DOI:** 10.1186/s43556-025-00369-1

**Published:** 2025-11-25

**Authors:** Gang Fan, Jinhui Zha, Shilin Chen, Pengxu Cang, Qingping Zhang, Jing Yang, Miao Liu

**Affiliations:** 1 Medical Research Center, Affiliated Nanshan Hospital of Shenzhen University, Shenzhen, 518052 China; 2 Department of Urology, The Second Affiliated Hospital of Guilin Medical University, Gulin, 541000 China; 3 Department of Endocrinology, Affiliated Nanshan Hospital of Shenzhen University, Shenzhen, 518052 China; 4 Department of Neurosurgery, Affiliated Nanshan Hospital of Shenzhen University, Shenzhen, 518052 China; 5 Department of Pathology, Brigham and Women’s Hospital, Harvard Medical School, Boston, MA 02115 USA

Colorectal cancer (CRC) remains a leading cause of cancer-related mortality worldwide, with current therapies like chemotherapy and targeted agents often limited by toxicity and resistance [1, 2]. Proprotein convertase subtilisin/kexin type 9 (PCSK9) is best known for regulating cholesterol by promoting the degradation of the LDL receptor (LDLR) in liver cells, which reduces LDL clearance and raises blood cholesterol levels [3]. However, emerging evidence has revealed that PCSK9 also plays a multifaceted role in cancer biology beyond lipid regulation, particularly in CRC progression and metastasis. This broader impact is largely attributed to PCSK9's ability to promote the degradation of various cell surface receptors beyond LDLR, including receptors involved in growth signaling, cell adhesion, and immune surveillance. By downregulating these key regulators, PCSK9 can directly influence oncogenic pathways.

Consequently, in CRC, PCSK9 contributes to tumor progression through various mechanisms, including modulation of lipid metabolism, promotion of epithelial-mesenchymal transition (EMT), and regulation of immune evasion [1]. Specifically, PCSK9 has been shown to enhance EMT by upregulating Snail1, downregulating E-cadherin, and activating PI3K/AKT signaling, thereby facilitating cancer cell migration and invasion. Additionally, PCSK9 influences the tumor microenvironment by promoting M2 macrophage polarization, further supporting tumor growth and metastasis [2, 4]. Thus, PCSK9 represents a promising therapeutic target that simultaneously influences multiple oncogenic processes within tumors.

In this study, we investigated the therapeutic potential of Cadd4 in colon cancer. We designed Cadd4 using computer-aided drug design (CADD), which included peptide optimization, molecular docking with PCSK9, and computational screening for PROTAC function, as previously described [3]. Originally developed to treat hypercholesterolemia, Cadd4 efficiently degrades PCSK9, restores LDLR expression, and reduces plasma cholesterol levels [3]. Now, it demonstrates broader utility. Our findings reveal its anti-tumor effects in colon cancer models, suppressing tumor growth by disrupting PCSK9-mediated signaling and inhibiting the PI3K-AKT pathway, marking the first evaluation of a PCSK9 degrader in cancer therapy.

In our in vitro experiments using rhodamine-labeled Cadd4, we confirmed its efficient cellular uptake in HCT116 and HT29 colon cancer cells, with fluorescence intensity increasing proportionally to both concentration (5–15 μM) and incubation time (1–6 h). Cadd4 treatment significantly reduced PCSK9 protein levels, with a DC50 of ~ 20 μM after 24 h. Time-course studies further showed progressive PCSK9 depletion, achieving significant degradation at 24 h (Data not shown). These results align with prior work in hepatic cells, where Cadd4 leveraged its peptide-based PROTAC design, combining a PCSK9-binding motif, E3 ligase-recruiting sequence (ALAPYIP), and a flexible linker, to promote ubiquitin–proteasome-dependent degradation [3].

In vivo efficacy was evaluated in nude mice bearing HCT116 xenografts. Mice received intraperitoneal injections of Cadd4 (15 mg/kg) or vehicle every other day for 14 days, starting when tumors reached 100–150 mm^3^. Fluorescence imaging of resected tumors confirmed Cadd4’s efficient tumor penetration(Data not shown). Cadd4 significantly suppressed tumor growth, it reduced tumor volume by > 70% and tumor weight by 50% compared to vehicle controls. Western blotting of resected tumors confirmed a ~ 75% reduction in PCSK9 protein levels (Fig. 1a), consistent with in vitro results (Data not shown).

**Fig. 1:**
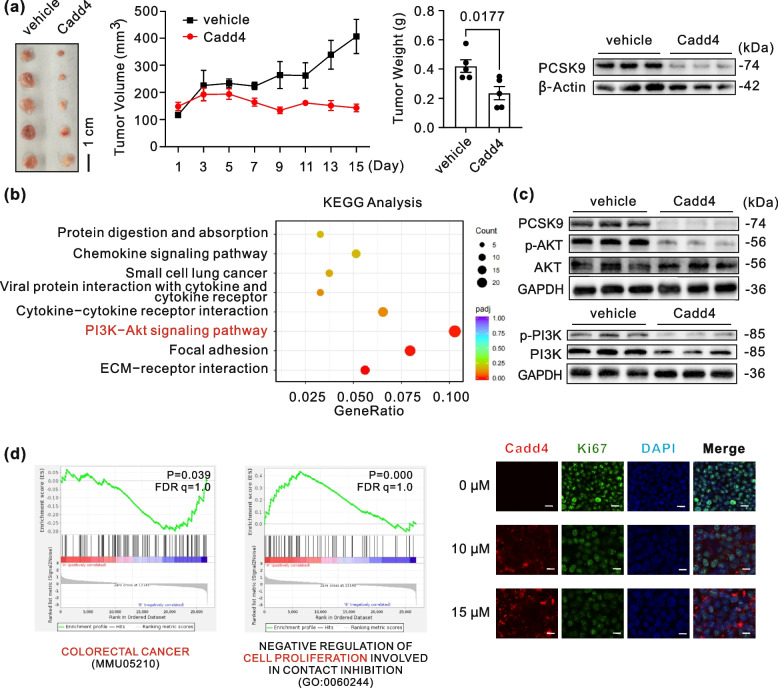
Cadd4 inhibits tumor growth and suppresses PI3K-signaling in HCT116 xenograft models. **a** Tumor volume and weight measurements over time in nude mice bearing HCT116 xenografts treated with vehicle or Cadd4 (N = 5; two-tailed unpaired Student’s t-test). Western blot analysis of PCSK9 expression in excised tumor tissues. β-Actin was used as a loading control. (N = 6; two-tailed unpaired Student’s t-test). **b** RNA sequencing analysis of tumor tissues following vehicle or Cadd4 treatment (N = 4). KEGG enrichment analysis of down-regulated genes. **c **Western blot analysis of PCSK9, PI3K, p-PI3K, AKT, and p-AKT protein expression in tumor lysates. GAPDH was used as a loading control (N = 5; two-tailed unpaired Student’s t-test). **d** Gene Set Enrichment Analysis (GSEA) of RNA-seq data from tumor tissues. (Left) Enrichment plot for the colorectal cancer pathway (MMU05210). (Right) Enrichment plot for negative regulation of cell proliferation in contact inhibition (GO:0060244). Immunofluorescence analysis of Ki67 expression in cells treated with Cadd4 (N = 5; two-tailed unpaired t-test)

To further elucidate the anti-tumor effects of Cadd4 in colon xenograft models, we conducted RNA sequencing (RNA-seq) on Cadd4-treated tumors. The KEGG enrichment analysis of down-regulated genes revealed significant pathways critical for cancer progression, including the PI3K-Akt signaling pathway (Fig. 1b). The PI3K-Akt pathway, frequently hyperactivated in colon cancer, promotes cell survival, angiogenesis, and chemoresistance [1, 2]. Western blotting further confirmed decreased phosphorylation of PI3K and Akt, key drivers of oncogenic signaling in vivo (Fig. 1c). Additionally, gene set enrichment analysis (GSEA) confirmed the suppression of colorectal cancer-associated genes (MMU05210) and proliferation-related terms (GO:0060244) (Fig. 1d). Immunofluorescence staining revealed a 60% reduction in Ki67-positive cells, indicating diminished proliferative activity (Fig. 1d).

The repurposing of Cadd4 from treating hypercholesterolemia to targeting colon cancer underscores the versatility of TPD technologies. Its efficacy in both in vitro and in vivo models makes it as a promising candidate for further development. Unlike antibodies that target extracellular PCSK9 [5], Cadd4 enters cancer cells to degrade intracellular PCSK9, offering a potential advantage over existing treatments. As a PROTAC, Cadd4 functions substoichiometrically, allowing for sustained target clearance at low doses and reducing the risk of resistance, which is advantageous over inhibitors that necessitate continuous high concentrations [3]. Cadd4 exhibits no hepatic toxicity or systemic adverse effects in mice [3]. In contrast, gene-editing approaches carry risks of off-target mutations and genotoxic risks. Moreover, Cadd4 is synthesized via solid-phase peptide synthesis (SPPS), a scalable, low-cost process compared to monoclonal antibodies or CRISPR [3].

Although Cadd4 showed no liver toxicity or weight changes in mice [3], long-term safety studies in cancer models are still needed. Future work should also investigate combinatorial therapies, as PI3K-Akt inhibition synergizes with chemotherapy or immunotherapy in preclinical studies. In this study, all in vivo efficacy trials were conducted using immunodeficient nude mouse models. Although this model was appropriate for evaluating the direct anti-tumor effects of Cadd4, it did not allow for the assessment of potential adaptive immune responses. Additionally, the anti-tumor effects of Cadd4 are likely multifaceted. While our focus in this study was on the PI3K-AKT pathway, future research should further investigate its impact on lipid metabolism, tumor immunity, and other mechanistic aspects.

In conclusion, Cadd4 represents a paradigm shift in colon cancer treatment, merging CADD precision with TPD’s transformative potential. By degrading PCSK9 and inhibiting PI3K-Akt signaling, it disrupts tumorigenic networks at multiple nodes. This study not only expands the therapeutic scope of Cadd4 but also highlights the untapped potential of repurposing metabolic regulators for oncology applications.

## Supplementary Information

Supplementary Material 1.

## Data Availability

All data supporting the findings of this study are available upon request by contacting the corresponding author.
