# Supplementary material for: Computer-aided design enables repurposing of proprotein convertase subtilisin/kexin type 9 degrader from cholesterol-lowering to colon cancer therapy
Source: Mol Biomed. 2025 Nov 25;6:120. doi: 10.1186/s43556-025-00369-1 (PMC12647423; doi:10.1186/s43556-025-00369-1)
Supplement: Supplementary file 1 — Supplementary Material 1. [file 43556_2025_369_MOESM1_ESM.docx]

**Computer-Aided Design Enables Repurposing of Proprotein**

**Convertase Subtilisin/kexin Type 9 Degrader from Cholesterol-Lowering to Colon Cancer Therapy**

Gang Fan^1^*, Jinhui Zha^1,2^*, Shilin Chen^3^*, Pengxu Cang^4^, Qingping Zhang^4^, Jing Yang^3#^, Miao Liu^5#^

1. Medical Research Center, Affiliated Nanshan Hospital of Shenzhen University, Shenzhen 518052, China
2. Department of Urology; The Second Affiliated Hospital of Guilin Medical University, Gulin, 541000, China
3. Department of Endocrinology, Affiliated Nanshan Hospital of Shenzhen University, Shenzhen 518052, China
4. Department of Neurosurgery, Affiliated Nanshan Hospital of Shenzhen University, Shenzhen 518052, China
5. Department of Pathology, Brigham and Women’s Hospital, Harvard Medical School, Boston, MA, United States

*These authors contributed equally.

**Corresponding authors:**

Miao Liu, MD & PhD,

Department of Pathology, Brigham and Women's Hospital

Harvard Medical School

Boston, MA 02115, United States

1. mail: [mliu0@bwh.harvard.edu](mailto:mliu0@bwh.harvard.edu)

Jing Yang, MD

Department of Endocrinology,

Affiliated Nanshan Hospital of Shenzhen University,

Shenzhen 518052, China

E-mail: jing.yang@uszh.org.cn

**Materials and Methods**

**Structural Prediction and Interface Validation**

We employed AlphaFold 3 (AF3) with its default general-purpose model to predict the three-dimensional structure of the protein-peptide complex. The model with the highest confidence score (pLDDT) was selected for subsequent analyses. To assess the physicochemical plausibility of the predicted interface, we performed energy-based evaluations using the Rosetta molecular modeling suite. The complex structure was first relaxed with the FastRelax protocol under the ref2015 scoring function to eliminate steric clashes and optimize side-chain conformations. Interaction energies across the interface were then calculated, and a detailed interaction map was generated using the AnalyzeComplex application.

**Animals**

A total of 6-week-old male nude mice were used in this study. All animals were housed in the specific pathogen-free (SPF) facility of the Animal Center at Shenzhen University Affiliated Nanshan Hospital. Mice were maintained in individually ventilated cages under standardized conditions, including a 12-hour light/dark cycle, with free access to autoclaved drinking water and a standard laboratory diet. At the end of the experiment, mice were deeply anesthetized via inhalation of isoflurane until cessation of breathing, followed by cervical dislocation and organ collection. All animal procedures were approved by the Institutional Animal Care and Use Committee of Shenzhen University Affiliated Nanshan Hospital and the Ethics Committee of TOPBIOTECH Shenzhen (Approval No.: TOP-IACUC-2023-0256). All experiments were conducted in accordance with relevant guidelines and regulations.

**Xenograft Models**

Xenograft models were generated as described previously [1]. Briefly, A total of 2–5 × 10^6^ cells suspended in 0.1 mL of PBS were subcutaneously injected into the bilateral dorsal flanks of 6-week-old female nude mice. Tumor dimensions were periodically measured using a caliper. Once tumor volumes reached 100-150 mm³, the mice (N = 5 per group) were randomly assigned to treatment groups. The groups included: (1) PBS (vehicle) control; (2) Cadd4 (15 mg/kg). Cadd4 or PBS (vehicle) was administered via intraperitoneal injection every two days. All compounds were prepared in PBS. Tumor volume was calculated according to the following formula: Tumor volume = [length × width × (length + width)/2] × 0.56 [2]. The maximum permitted tumor size was 1.25 cm in any dimension, which was not exceeded during the study.

**Cell Culture**

The human colon cancer cell lines HCT116 and HT-29 were acquired from the China Center for Type Culture Collection (CCTCC, Shanghai, China). Cells were maintained in McCoy’s 5A medium (Procell Life Science & Technology Co., Ltd.) supplemented with 10% fetal bovine serum (FBS; Gibco, Thermo Fisher Scientific) and 1% penicillin–streptomycin (Beyotime Biotechnology Co., Ltd.), at 37 °C in a humidified atmosphere containing 5% CO2. All cell lines were authenticated using short tandem repeat (STR) profiling and routinely tested to confirm the absence of mycoplasma contamination.

**Antibodies and Reagents**

The following antibodies and reagents were used for western blot (WB) and immunofluorescence (IF) analyses. Details regarding catalog numbers, suppliers, and dilutions are summarized below: PCSK9 (WB): Rabbit polyclonal antibody (Cat. #A00085-2, BOSTER; 1:1000); GAPDH (WB): Rabbit monoclonal antibody (Cat. #AF1186, Beyotime; 1:3000); β-Actin (WB): Rabbit polyclonal antibody (Cat. #bs-0061R, Bioss; 1:3000); PI3 Kinase (WB): Rabbit polyclonal antibody (Cat. #R22768, Zenbio; 1:750); Phospho-PI3 Kinase (WB): Rabbit polyclonal antibody (Cat. #341468, Zenbio; 1:750); AKT (WB): Rabbit polyclonal antibody (Cat. #R23412, Zenbio; 1:750); Phospho-AKT (WB): Rabbit polyclonal antibody (Cat. #R22961, Zenbio; 1:750); Phospho-EIF4EBP1 (Thr37/46) (WB): Rabbit polyclonal antibody (Cat. #AF5806, Beyotime; 1:10000); Ki-67 (IF): Rabbit polyclonal antibody (Cat. #27309-1-AP, Proteintech; 1:100); Goat Anti-Rabbit IgG H&L (Cat. #111-035-144, Jackson; 1:10000); Goat Anti-Rabbit IgG H&L (Alexa Fluor® 647) (Cat. #ab150083, Abcam; 1:400); Goat Anti-Rabbit IgG H&L (Alexa Fluor® 488) (Cat. #ab150077, Abcam; 1:400); 180 kDa Plus Prestained Protein Marker(Cat. #MP201-01, Vazyme; 1:400); Prestained Protein Marker IV (8-200 kDa) (Cat. #G2083-250UL, Servicebio; 1:400).

**Western Blot Analysis**

Western blotting was carried out according to a previously established protocol from our laboratory [3]. Briefly, samples were washed with cold phosphate-buffered saline (PBS) and lysed using RIPA buffer containing a protease and phosphatase inhibitor cocktail. Protein concentrations were quantified, and equal amounts of lysates were separated by SDS-PAGE and subsequently transferred to PVDF membranes. The membranes were blocked and then incubated with specific primary antibodies overnight at 4 °C, followed by incubation with horseradish peroxidase (HRP)-conjugated secondary antibodies. Protein bands were visualized using an enhanced chemiluminescence (ECL) detection system. Quantification of target proteins was performed using ImageJ software.

**Biodistribution of Cadd4 by In Vivo Imaging System**

Biodistribution analysis using vivo imaging system was been described previously [4]. The rhodamine-labeled Cadd4 were intraperitoneal injected into the nude mice. The mice were anesthetized with 2% isoflurane, and their distribution was monitored at pointed 4h, by an imaging system (InSyTe FLECT/CT). After euthanasia of the mice, the dissected tumor were collected for ex vivo imaging.

**Statistical analysis**

All quantitative data are presented as mean ± standard deviation (SD). The number of biological replicates (n) is indicated in each figure legend. Normality was assessed using the Shapiro–Wilk test. For comparisons between two groups, an unpaired two-tailed Student’s t-test was used. For multiple comparisons across groups, one-way or two-way ANOVA followed by Tukey’s honestly significant difference test was applied, depending on the experimental design. Statistical analyses were conducted using GraphPad Prism software (version 9.5). Differences were considered statistically significant at p < 0.05.

**Reference**

1. Hidalgo M, Amant F, Biankin AV, Budinská E, Byrne AT, Caldas C, et al. Patient-derived xenograft models: an emerging platform for translational cancer research. Cancer Discov. 2014;4(9):998-1013.
2. Tomayko MM, Reynolds CP. Determination of subcutaneous tumor size in athymic (nude) mice. Cancer Chemother Pharmacol. 1989;24:148–154.
3. Luo QT, Ye YC, Guo WM, Zhu Q, Wang SS, Li N, et al. Senolytic Treatment Improve Small Intestine Regeneration in Aging. Aging Dis. 2024;15(4):1499–1507.
4. Fan G, Lu JF, Tan RR, Guo WM, Hong L, Zha JH, et al. Potent Efficacy of Computer-Aided Designed Peptide Degrader Drug on PCSK9-Mediated Hypercholesterolemia. bioRxiv. 2025;06.25:661251.
